# The evaluation of *Staphylococcus aureus* and *Staphylococcus epidermidis* in hospital air, their antibiotic resistance and sensitivity of *S. aureus* to cefoxitin

**DOI:** 10.1038/s41598-024-59463-z

**Published:** 2024-04-22

**Authors:** Mostafa Leili, Sedighe Afrasiabi, Roohollah Rostami, Mohammad Khazaei, Mahdaneh Roshani, Zahra Tarin

**Affiliations:** 1 Department of Environmental Health Engineering, School of Public Health and Research Center for Health Sciences, Hamadan University of Medical Sciences, Hamadan, Iran; 2 Department of Environmental Health Engineering, School of Public Health, Student Research Committee, Hamadan University of Medical Sciences, Hamadan, Iran; 3 Department of Environmental Health Engineering, Research Center for Health Sciences and Technologies, Semnan University of Medical Sciences, Semnan, Iran; 4 Department of Microbiology, Faculty of Medicine, Medical Microbiology, Hamadan University of Medical Sciences, Hamadan, Iran; 5 Department of Occupational Health Engineering, School of Public Health, Hamadan University of Medical Sciences, Hamadan, Iran

**Keywords:** Hospital air, Cefoxitin antibiogram (FOX), *Staphylococcus aureus*, *Staphylococcus epidermidis*, Microbiology, Health care

## Abstract

Staphylococci as a nosocomial infection agent, increases the possibility of contracting diseases such as wound infection, sepsis and skin infections in humans. It was shown that *Staphylococcus aureus* considered as a commensal organism causing various both endemic and epidemic hospital-acquired infections. Air samples were collected from Sina Hospital, Hamadan city, which dedicated to various respiratory diseases and analysed by biochemical tests. The resistance and sensitivity of bacterial strains to the cefoxitin antibiotic were also determined. *Staphylococcus aureus* density (CFU/m^3^) were measured in the air of various wards as follows: infectious 13.35 ± 7.57, poisoning 29.84 ± 33.43, emergency 8.64 ± 2.72, eye operation room 0, recovery room 6.28 ± 4.90, skin outpatient operation room 4.71 ± 2.36, respiratory isolation 0, ICU 0.79 ± 1.36, and the administrative room 6.28 ± 5.93; while the *Staphylococcus epidermidis* were as follows: infectious 1.57 ± 2.35, poisoning 2.35 ± 4.08, emergency 2.35 ± 2.35, eye operation room 0, recovery room 0.78 ± 1.36, skin outpatient operation room 2.35 ± 2.35, respiratory isolation 0, ICU 2.35 ± 4.08, and the administrative room 1.57 ± 1.36. The positive and negative control samples showed a concentration of 0. Moreover, among the *S. aureus* isolates, 33.3% were found to be resistant to cefoxitin, while 40.6% showed to be sensitive. Based on the results, the number of active people and the type and quality of ventilation are very effective in the air quality of various wards of hospital. The poisoning section showed the most contaminated air and the highest resistance and sensitivity to the cefoxitin antibiotic.

## Introduction

Bioaerosols represent the largest group of pollutants that can cause a significant number of patient deaths annually due to hospital-acquired infections^1^. Patients who contract hospital-acquired infections not only experience financial and psychological challenges but also place a substantial burden on governments in terms of financial and facilities. Hospital infections can occur within a range of 2–10 days after hospitalization, up to 30 days after surgery, or within 3 days after discharge. Individuals with weakened immune systems are especially vulnerable to these infections^1–5^. In 2017, the World Health Organization (WHO) introduced *Staphylococcus aureus* as one of the major infectious bacteria in hospitals. This bacterium can cause a range of diseases, from minor infections to death^4^. In addition to toxin production within the host, pathogenic bacteria have the ability to inhibit protein synthesis and trigger immune responses in individuals, while these bacteria can cause damage to cell membranes and cell walls, resulting in respiratory inflammation, asthma, bronchitis, and byssinosis. The release of pathogenic germs may impact children, the elderly, chronic patients with compromised immune systems, healthcare providers^5^. Various studies have indicated that antibiotic-resistant methicillin strains are frequently identified as a common cause of infections in operating rooms and postoperative settings^6,7^. According to the 2022 Clinical and Laboratory Standards Institute, cefoxitin antibiotics have been replaced with methicillin^8^.

If air quality of hospitals is not properly managed, diseases can spread throughout the hospital. Therefore, adequate ventilation plays a crucial role in maintaining a healthy environment. These assessments were taken to gain insights into the microbial contamination and potential antibiotic resistance in the hospital's air. Thus, this study aimed at assess the microbial (including *Staphylococcus aureus* and *Staphylococcus epidermidis*) quality of the air in various ward of Sina Hospital, which dedicated to various respiratory diseases, Hamadan city, and investigating the resistance and sensitivity of these bacteria to the cefoxitin (FOX).

## Materials and methods

### Study design

The present study is a cross-sectional. The wards/rooms included in the study are poisoning, infectious, internal, eye operating, skin outpatient operating, operation recovery, emergency, administrative, ICU, and respiratory isolation.

### Site selection

In this study, sampling locations were selected based on the type of ventilation and the number of active people in each ward. Sampling sites were stratified into wards with positive or negative pressure enhanced ventilation, with/without HEPA filters and UV lamps, as well as conventional ventilation with hospitalized patients and a non-hospitalized office ward, along with a negative and positive control samples taken from the low density area of the hospital yard^9^.

### Data collection

Microbial sampling was performed according to standard protocols. Active sampling was conducted using a Quick Take 30 pump (SCK company, UK) with a microvacuum cassette, for 15 min at a flow rate of 28.3 L/min, that repeated three times during the morning and evening shifts. The sampling height and distance from walls were based on a similar study^10^. The sampling device was set at a height of 120–150 cm from the ground (within the breathing area) and at a distance of more than 1 m from walls and obstacles. At the same time, air temperature was recorded using a thermometer, and air humidity was measured with a hygrometer. Additionally, the volume of air moved per hour was calculated by determining the air inlet and outlet speed with a thermal anemometer^10,11^. The general characteristics (conditions) of the studied sections is shown in the Table 1. Based on this table, the distribution of people in the studied sections/wards of the hospital were also determined.

**Table 1:** General characteristics of the sampling site.

The name of the ward	Number of rooms	The average number of people during sampling	Average full/empty beds	Number of windows (open/closed)	Window area (m^2^)	Number of doors (open/closed)	Room volume (m^3^)
Infectious	4	34	4 (F)/33 (E*)	4 (C*)	1.08	1 (O)	90
Internal	11	37	4 (F)/7 (E)	1(O*^)^/3 (C)	0.05	1 (O)	90
Poisoning	7	24	67 (F)/34 (E)	1 (O)/3 (C)	1.08	1 (O)	72
Official	13	26	–	2 C	0.99	1 (O)	54
Emergency	1	17	6 (F)/16 (E)	2 (O)/6 (C)	0.99	1 (O)	250
Eye operating room	3	10	1 (F)/1 (E)	–	–	–	105
Dermatology outpatient operating room	1	34	3 (F)/2 (E)	4 (C)	0.99	1 (O)	147
Respiratory isolation	1	8	4 (F)/67 (E)	4 (C)	0.99	–	195
Eye surgery recovery	1	35	4 (F)/7 (E)	–	–	–	72
ICU	1	26	7 (F)/7 (E)	8 (C)	0.99	1 (O)	350

*O Open, *C* Closed, *F* Full, *E* Empty.

### Samples and biochemical tests

In total, the collected samples were identified by differential methods including Gram staining, and biochemical tests such as mannitol, catalase, coagulase, DNase agar, hemolysis type, and novobiocin sensitivity^10,12–15^. The disc diffusion method was used to identify the resistant and sensitive strains of *Staphylococcus aureus* bacteria in the air in relation to the cefoxitin antibiotic. The diameter of the resulting zones was measured and compared with the standard table provided by the Clinical and Laboratory Standards Institute^8^.

### Statistical analyses

The data were analyzed using SPSS 26 software. The normal and non-normal distributions of *Staphylococcus aureus* and *Staphylococcus epidermidis* bacteria in the air of poorly ventilated, conventional, and advanced hospital wards were investigated using the Kolmogorov–Smirnov and Shapiro–Wilk tests. Following these investigations, the data from the normal distribution of *S. aureus* in conventionally ventilated wards (internal, poisoning, infectious) and advanced ventilation wards (outpatient skin operating room) and administrative wards were analyzed through ANOVA and post-hoc tests (Tukey's HSD, Scheffe, and LSD).

Additionally, the normal distribution of *S. epidermidis* in advanced ventilated wards (respiratory isolation and eye operating room) and conventionally ventilated wards (emergency and infectious) was evaluated using the aforementioned tests. The non-normal distribution of *S. aureus* in the ICU and emergency wards was investigated through the Mann–Whitney test. The non-normal distribution of *S. epidermidis* in conventionally ventilated wards (poisoning and internal), the administrative ward, and advanced ventilated wards (ICU and skin outpatient operating room) was investigated using the Kruskal–Wallis test. Furthermore, the homogeneity of variances was analyzed based on the mean for both bacteria according to Levene's test**.**

## Results

### Density, frequency distribution and sensitivity analysis

**Figure 1:**
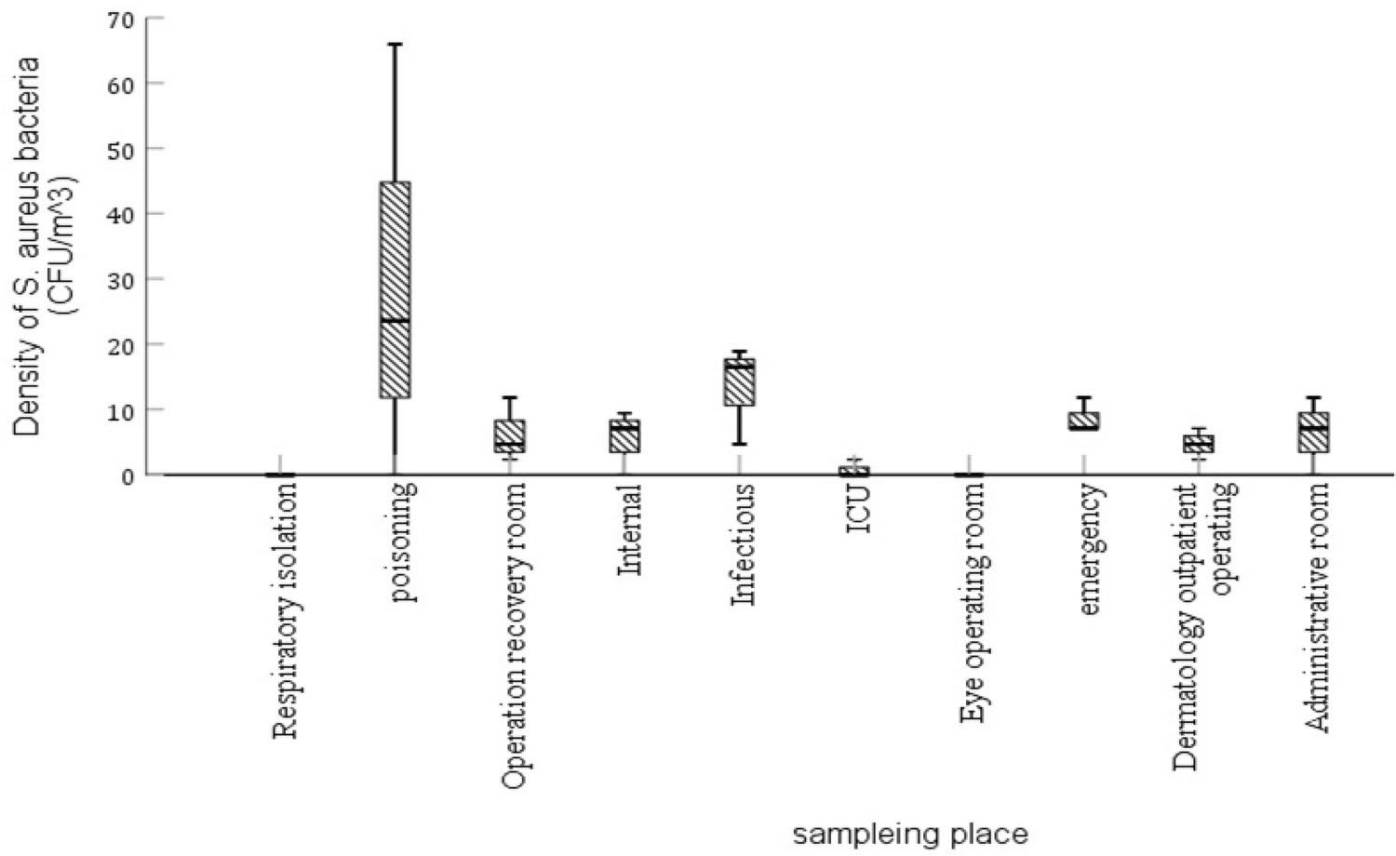
The distribution of *S. aureus* found in the air of various wards.

This study aimed to measure the microbiological quality of the air in Sina Hospital. The frequency distribution of *S. aureus* bacterium sampled from the air of various hospital wards is shown in Fig. 1.

**Figure 2:**
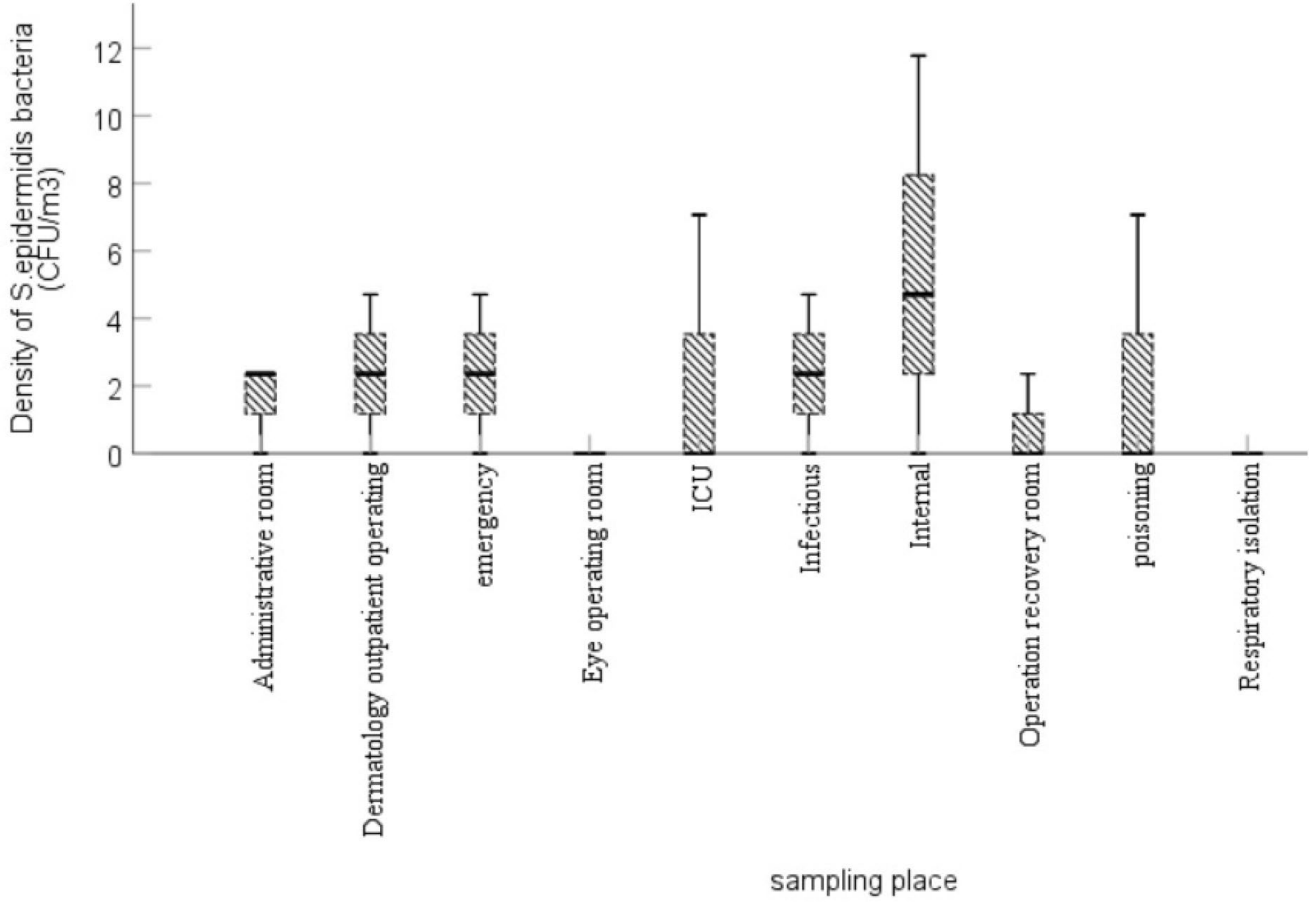
The distribution of *S. epidermidis* found in the air of various wards.

According to Fig. 1, the distribution of *S. aureus* bacterium from a total of 96 colonies identified from the air of different hospital wards are as follows (CFU/m^3^): infectious 13.35 ± 7.57, poisoning 33.43 ± 29.84, internal 4.90 ± 5.50, emergency 2.72 ± 8.64, eye operating 0, recovery 4.90 ± 6.28, skin outpatient operating 0.28 ± 4.71, respiratory isolation 33.43 ± 0.79, ICU 4.90 ± 0.79, and administrative 1.36 ± 0.79. Similarly, the frequency distribution of *S. epidermidis* bacterium in the air of different wards is shown in Fig. 2.

A total of 25 colonies of *S. epidermidis* bacterium were detected in the air of various hospital wards. The mean and standard deviation values (CFU/m^3^) are as follows: infectious 35.12 ± 2.10, poisoning 4.08 ± 2.35, internal 5.92 ± 5.42, emergency 2.35 ± 2.30, eye operation 0, operation recovery 1.36 ± 0.78, respiratory isolation 2.35 ± 2.35, ICU 4.08 ± 2.35, and administrative room 1.36 ± 1.58.

**Figure 3:**
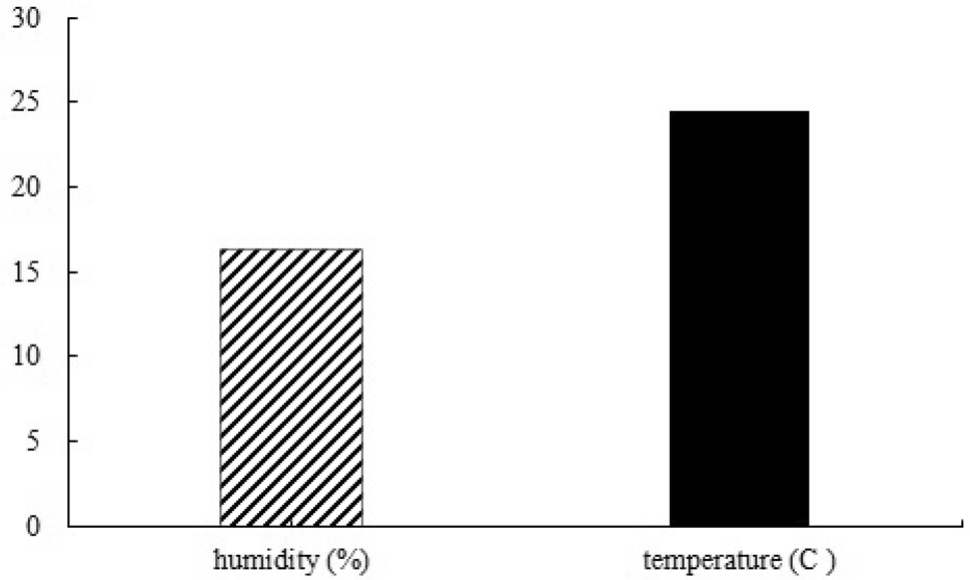
The average temperature (°C) and air humidity (%) levels in the studied wards.

Changes in temperature and humidity of the various sampling wards/sites during the sampling period as the main contributing factors are shown in Fig. 3.

According to the Fig. 3, the average temperature ranged from 28.50 °C in the emergency to 20.60 °C in the eye operating room. Additionally, the highest humidity level was recorded in the respiratory isolation room at 24%, while the administrative room had the lowest humidity level of 11%.

**Figure 4:**
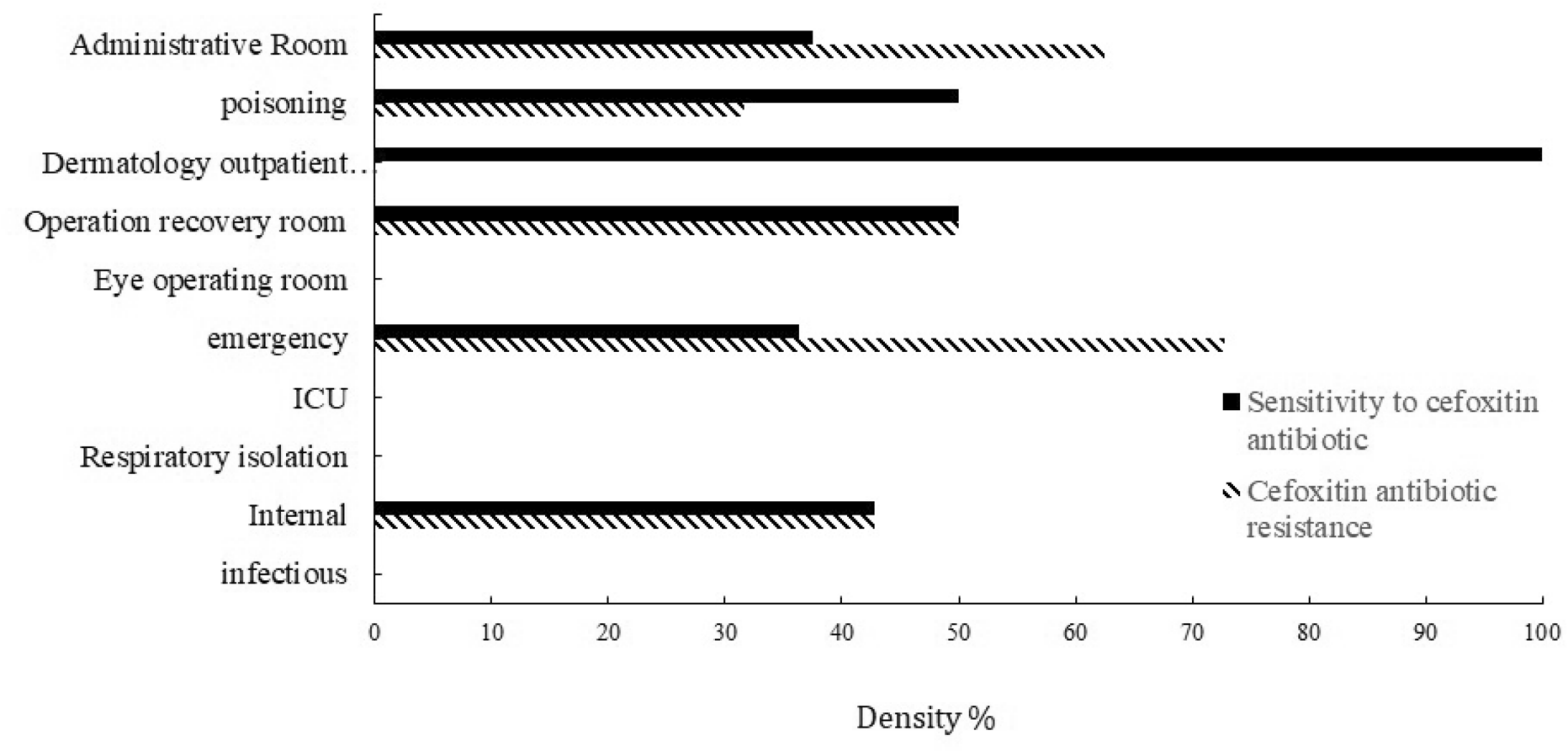
The density of *S. aureus* strains resistant and sensitive to the cefoxitin antibiotic in the air of hospital wards.

The frequency distribution of resistant and sensitive strains of *S. aureus* in the air in different wards of the hospital with the cefoxitin antibiogram (FOX) is shown in Fig. 4.

Figure 4 shows that *S. aureus* bacterium were resistant and sensitive to the cefoxitin antibiogram disc (FOX), with a mean and standard deviation of 6.27 ± 9.79 (CFU/m^3^) and 7.65 ± 13.48, respectively. Among the sampling sites, the emergency ward had the highest percentage (75%) of *Staphylococcus aureus* samples resistant to the cefoxitin antibiotic disk. In contrast, the infectious ward, respiratory isolation ward, eye operating room, and ICU had no resistant samples. While, *Staphylococcus aureus* sensitive to the cefoxitin antibiotic disk was detected in 100% of the samples taken from the skin outpatient operating room. However, it was not observed (0%) in samples from the infectious ward, respiratory isolation ward, eye operating room, and ICU.

### The number of air changes per hour

To determine the quality of air resulting from mechanical ventilation, we calculated the number of air changes in the respiratory isolation room and the eye operating room according to the ACGIH (American Conference of Governmental Industrial Hygienists) standard, as reported in Table 2.

**Table 2:** The calculation of the flow rate of blowers and suction in the respiratory isolation room.

		Blower			Suction			
		B 1	B 2	B 3	S 1	S 2	S 3	S 4
Dimensions (cm)	Length	40	40	40	40	40	40	40
	Width	40	40	40	40	40	40	40
Area (cm^2^)		1600	1600	1600	1600	1600	1600	1600
Area in (F^2^)		1.80	1.80	1.80	1.80	1.80	1.80	1.80
Speed (FPS)		1.33	3.15	0.15	4.80	4.40	1.73	1.80
Flow rate (CFM)		137.25	325.07	15.48	495.35	454.07	178.53	185.76
Total flow rate (CFM)		477.81			1313.71			
Number of air changes per hour					11.45			

According to the ACGIH standard, the number of air changes per hour is calculated by selecting the higher flow rate between the blowers and suctions shown in Table 2, using the total suction flow rate. The air changes per hour were calculated to be 11.45 (Eq. 1).

1 $$\mathrm{Air\,change\,per\,hour}=\frac{60\times {\text{Q}}}{{\text{V}}}$$

where, Q stands for the flow rate, and V stands for the volume of the sampling ward/room.

According to the standards of the American Conference of Health Professionals, the performance of this ventilation system exceeds 10 and 15 (as the standard values) air changes per hour for a respiratory isolation and an eye operating rooms, respectively^16^. Table 3 also report such information related to the eye operating ward.

**Table 3:** Calculating the flow rate of blowers and suction in the eye operating room.

		Blower				Suction			
		B 1	B 2	B 3	B 4	S 1	S 2	S 3	S 4
Dimensions (cm)	Length	40	40	40	40	40	40	40	40
	Width	40	40	40	40	40	40	40	40
Area (cm^2^)		1600	1600	1600	1600	1600	1600	1600	1600
Area in (F^2^)		1.72	1.72	1.72	1.72	1.72	1.72	1.72	1.72
Speed (FPS)		3.29	2.00	2.40	5.10	0.14	0.69	0.27	0.19
Flow rate (CFM)		339.53	206.40	247.67	526.31	14.45	17.20	28.86	19.61
Total flow rate (CFM)		1319.90				133.12			
Number of air changes per hour						21.34			

The air exchange rate for the eye surgery ward was calculated to be 21 times per hour, which is higher than the standard rate determined by the American Health Professionals Conference, showing the acceptable performance of the ventilation system.

### Data analysis

According to the Kolmogorov–Smirnov and Shapiro–Wilk tests, the distribution of *S. aureus* in the rooms with advanced ventilation and conventional ventilation was found to be normal (p-value < 0.05). However, the distribution of this bacterium in the administrative ward was found to be non-normal. Additionally, the distribution of *S. epidermidis* was observed to be non-normal in all groups, including those with advanced ventilation, conventional ventilation, and administrative ward ventilation. In addition, the average and standard deviation of *S. aureus* in the group with improved ventilation system (including eye operating, respiratory isolation, and the ICU rooms) were calculated to be 0, 0, 1.36 ± 0.79 (CFU/m^3^), respectively; and in the group with conventional ventilation (including internal, infectious, emergency, poisoning, a skin outpatient operating, and operating recovery rooms) were calculated to be 5.50 ± 4.90, 13.35 ± 7.57, 8.64 ± 2.72, 29.84 ± 33.43, 4.71 ± 2.36, and 6.28 ± 4.90 (CFU/m^3^), respectively; and in the administrative room, were reported to be 6.28 ± 5.93 (CFU/m^3^). Using the ANOVA test, a significant difference (p-value < 0.05) was observed in the distribution of *S. aureus* among the infectious, internal, poisoning, skin outpatient, recovery, and administrative rooms. However, normal distribution of *S. epidermidis* was not observed in the emergency rooms, outpatient operating rooms, and internal operating rooms. Using the Mann–Whitney test, a statistically significant differences (p-value < 0.05) was found in the non-normal distribution of *S. aureus* in the emergency room and ICU. However, the non-normal distribution of *S. epidermidis* in the air of ICU rooms, recovery rooms, and poisoning did not show a significant differences using the Kruskal–Wallis test.

## Discussion

Nosocomial infections are transmitted through pathogenic bioaerosols. As a result, hospitals are more likely to face the risk of infections, with hundreds of millions of people falling ill each year^5,17,18^. 70% of the identified bacteria were found to be resistant to antibiotics, with resistant strains of *Staphylococcus aureus* and *Staphylococcus epidermidis* being the most common^19^. In the current study, the air of the poisoning, infectious disease, and emergency wards were recorded as the most contaminated with *Staphylococcus aureus* due to poor ventilation and the high number of hospitalized patients, compared to the air of other studied wards. While *Staphylococcus epidermidis* detected in the air of internal wards, poisoning wards, and the ICU showed the highest density compared to other wards; however, the density of *Staphylococcus aureus* bacterium had a higher percentage compared to the density of *Staphylococcus epidermidis* bacterium in all assessed wards. According to the results, the distribution of bacteria did not follow a specific pattern, but the distribution of bacteria among the sections was statistically significant (p-value < 0.05). It was a function of the average number of active people in the hospital and ventilation performance in the wards. Additionally, the lack of bacterial growth during the sampling times in the air samples from the respiratory isolation sections and the eye operating room indicated the proper functioning of the mechanical ventilation systems. In the disc diffusion method, using cefoxitin antibiotic discs, the highest concentration of resistant strains was detected in the air of the emergency ward, while the skin outpatient operating room had the highest concentration of sensitive strains. This is likely due to poor ventilation and the diversity of diseases in the emergency room, and the concentration of skin diseases in the skin outpatient operating room, respectively. Also, based on the results of similar literature, the concentration of *Staphylococcus aureus* in the air of hospital wards has a direct relationship with the rate of infection in patients. According to the results of the present study, the distribution of bacteria differs in the air of different wards, and it was statistically significant (p < 0.05) regard to the rate of patients with infection diseases. This finding was consistent with the results of Claire E. Adams and colleagues^20^, that assessed the transmission of *Staphylococcus aureus* in the air and surfaces of the intensive care unit (ICU) and delivery rooms. Also, our findings are consistent with the results of Chemere Madebo et al.,'s study, regarding the relationship between the distribution of *Staphylococcus aureus* in the air of ICU wards and operating rooms of hospitals in southern Ethiopia with hospital infections^20,21^.

In the present study, bioaerosol sampling was done by an active method using the Quick take 30, which shows the air quality of the hospital in a short period of time by passing the air through the culture medium and trapping the bacteria, while the results obtained by the study of Girma et al., which was carried out with the same purpose of the present study, but using a passive method for sampling, showed a significant increase in the density of bacteria and was not consistent with the present study results, that could be due to the long sampling time, which in turn, resulted in the increasing of the possibility of error and false results^22^.

In this study, we examined the air quality of hospital wards regarding the presence of common bacteria such as *Staphylococcus aureus* and epidermidis, which can be used as an indicator of air pollution in the hospital environment. In addition, the results of Yuni et al.,'s study during the investigation of the microbial quality of the air in Kudus Hospital in Indonesia, showed that it is consistent with the present study in terms of the distribution of such bacteria^23^. Moreover, in the present study, the results showed the highest average of resistant *Staphylococcus aureus* bacterium at a rate of 33.3% compared to the antibiotic cefoxitin in the air of the emergency ward, which was logically observed based on the variety of patients in this ward. As well as the highest average of sensitive strains of these bacteria increased by 40.6% compared to the antibiotic cefoxitin in the air of the skin outpatient operating room due to the lack of appropriate ventilation and the presence of skin patients which are the same with the results of Almanaa and Colleagues results that have been reported in clinical samples^24^.

## Limitations and strengths of the study

### Limitations

The lack of sufficient funds and facilities for sampling throughout the year can be considered as a main limitation. Also, due to the requirements of the hospital's health unit during sampling, the opportunity to take samples during the night shift was excluded from the current study**.**

### Strengths

One of the main strengths of this study is determining the amount of resistant and sensitive strains of bacteria in the hospital air. Moreover, considering the importance of ventilation for air quality, and the direct relationship between the pathogenicity of these bacterial strains and their impact on exposed individuals, these correlations were assessed and this goal motivated our investigation.

## Conclusions

In the present study, the relationship between the density of airborne bacteria and resistant and sensitive strains in different wards of the hospital was investigated. According to the results, the air samples from the poisoning ward showed the highest contamination with *S. aureus* and *S. epidermidis*. The emergency room showed the most resistant strains of *S. aureus* to cefoxitin antibiogram (FOX), whereas the outpatient skin operating ward showed the most sensitive strains. Therefore, a high bacterial concentration does not necessarily indicate an increase in the concentration of resistant and sensitive strains. Interestingly, the operating rooms for eye surgeries and respiratory isolation areas were found to be free of contamination, indicating the effective functioning of the mechanical ventilation system of these wards according to respective standards.

## Data Availability

The authors declare that the data supporting the findings of this study are available within the paper. Should any raw data files be needed in another format they are available from the corresponding author upon reasonable request. All sampling and analysing methods conducted according to conventional valid procedures/protocols as cited in “Materials and methods” section.
